# Contributions of Function-Altering Variants in Genes Implicated in Pubertal Timing and Body Mass for Self-Limited Delayed Puberty

**DOI:** 10.1210/jc.2017-02147

**Published:** 2017-11-16

**Authors:** Sasha R. Howard, Leonardo Guasti, Ariel Poliandri, Alessia David, Claudia P. Cabrera, Michael R. Barnes, Karoliina Wehkalampi, Stephen O’Rahilly, Catherine E. Aiken, Anthony P. Coll, Marcella Ma, Debra Rimmington, Giles S. H. Yeo, Leo Dunkel

**Affiliations:** 1 Centre for Endocrinology, William Harvey Research Institute, Barts and the London School of Medicine and Dentistry, Queen Mary University of London, London EC1M 6BQ, United Kingdom; 2 Centre for Integrative Systems Biology and Bioinformatics, Department of Life Sciences, Imperial College London, London SW7 2AZ, United Kingdom; 3 Centre for Translational Bioinformatics, William Harvey Research Institute, Barts and the London School of Medicine and Dentistry, Queen Mary University of London, London EC1M 6BQ, United Kingdom; 4 National Institute for Health Research Barts Cardiovascular Biomedical Research Unit, Queen Mary University of London, London EC1M 6BQ, United Kingdom; 5 Children’s Hospital, Helsinki University Hospital and University of Helsinki, FIN-00029 HUS Helsinki, Finland; 6 University of Cambridge Metabolic Research Laboratories and Medical Research Council Metabolic Diseases Unit, Wellcome Trust-MRC Institute of Metabolic Science, Addenbrooke’s Hospital, Cambridge CB2 0QQ, United Kingdom; 7 Department of Obstetrics and Gynaecology, University of Cambridge, Cambridge CB2 0SW, United Kingdom; 8 National Institute for Health Research, Cambridge Comprehensive Biomedical Research Centre, Cambridge CB2 0SW, United Kingdom

## Abstract

**Context:**

Self-limited delayed puberty (DP) is often associated with a delay in physical maturation, but although highly heritable the causal genetic factors remain elusive. Genome-wide association studies of the timing of puberty have identified multiple loci for age at menarche in females and voice break in males, particularly in pathways controlling energy balance.

**Objective/Main Outcome Measures:**

We sought to assess the contribution of rare variants in such genes to the phenotype of familial DP.

**Design/Patients:**

We performed whole-exome sequencing in 67 pedigrees (125 individuals with DP and 35 unaffected controls) from our unique cohort of familial self-limited DP. Using a whole-exome sequencing filtering pipeline one candidate gene [fat mass and obesity–associated gene (*FTO*)] was identified. *In silico*, *in vitro*, and mouse model studies were performed to investigate the pathogenicity of *FTO* variants and timing of puberty in *FTO^+/−^* mice.

**Results:**

We identified potentially pathogenic, rare variants in genes in linkage disequilibrium with genome-wide association studies of age at menarche loci in 283 genes. Of these, five genes were implicated in the control of body mass. After filtering for segregation with trait, one candidate, *FTO*, was retained. Two *FTO* variants, found in 14 affected individuals from three families, were also associated with leanness in these patients with DP. One variant (p.Leu44Val) demonstrated altered demethylation activity of the mutant protein *in vitro*. *Fto*^+/−^ mice displayed a significantly delayed timing of pubertal onset (*P* < 0.05).

**Conclusions:**

Mutations in genes implicated in body mass and timing of puberty in the general population may contribute to the pathogenesis of self-limited DP.

## Teaser

We assessed the contribution of rare variants in genes important in the timing of puberty and body mass regulation in the general population to the phenotype of familial delayed puberty.

Puberty is the maturational process of the reproductive endocrine system that results in adult height and body proportion, in addition to the capacity to reproduce. A minimum level of energy availability is required for the onset of puberty, whereas increased fat mass has been shown to be associated with precocious onset of puberty (1, 2). However, a role for genes connected with regulation of body mass have not been clearly demonstrated in pubertal timing.

The existence of genetic heterogeneity in pubertal timing is supported by several large genome-wide association studies (GWAS) of the age at menarche (AAM) (3–5). Evidence (*P* < 5 × 10^−8^) for 123 signals at 106 genomic loci has been identified. Many of these loci were associated with Tanner staging in both sexes, suggesting that these data are applicable to both men and women (6, 7).

The first of many GWAS loci associated with AAM was the developmental gene *LIN28B* (3, 8). Additional signals in genes involved in energy homeostasis and growth have been found near *LEPR-LEPROT*, which encodes the leptin receptor. Leptin (a key regulator of body mass) is an important permissive signal for the onset of puberty (9). In addition to leptin signaling, overlap with several genes implicated in body mass index was found, including fat mass and obesity–associated gene (*FTO*), *SEC16B*, *TMEM18*, and *NEGR1* (Supplemental Table 1) (5). Whether such genes may regulate pubertal timing exclusively via impact on fat mass or via other body mass index (BMI)–independent mechanisms is unknown (10).

Disordered pubertal timing affects up to 5% of adolescents and is associated with adverse health and psychosocial outcomes (11–14). Self-limited delayed puberty (DP) represents the extreme end of normal pubertal timing and is defined as the absence of testicular enlargement in boys or breast development in girls at an age that is 2 to 2.5 standard deviations (SD) later than the population mean (3). DP may be an isolated feature of the condition or be associated with constitutional delay in growth that can manifest from early childhood.

DP segregates within families, usually with an autosomal-dominant pattern of inheritance (15, 16). Despite strong heritability in most cases the genetic basis of DP remains elusive (17, 18). Moreover, the relevance of genetic factors influencing timing of puberty in the general population to patients with extreme pubertal delay has not been explored. Given the importance of energy balance for reproductive health, genes identified by AAM GWAS that relate to energy homeostasis are of particular interest. Our multigenerational DP families provide a highly valuable resource to investigate these candidate genes in familial DP.

## Materials and Methods

### Patients

The patients selected for this study were taken from a previously described, accurately phenotyped and characterized, Finnish DP patient cohort (19). Diagnosis is based on objective evidence of a delayed pubertal growth spurt rather than self-recall. Patients referred with DP to specialist pediatric care in central and southern Finland (1982 to 2004) were identified. All patients (n = 492) met the diagnostic criteria for self-limited DP, defined as the onset of Tanner genital stage II (testicular volume > 3 mL) > 13.5 years in boys or Tanner breast stage II > 13.0 years in girls (*i.e.*, 2 SD later than average pubertal development) (18, 20). Pubertal growth spurt in probands was >2 SD later than average age at acceleration of pubertal growth (take-off) beyond 13.8 and 12.2 years and age at peak height velocity (PHV) later than 15.6 and 13.7 years in males and females, respectively (21).

Chronic illness and undernutrition was excluded by medical history, clinical examination, and routine laboratory tests. Hypogonadotropic hypogonadism, if suspected, was excluded by spontaneous pubertal development at follow-up. In the 50% of patients who choose to have pubertal induction via the use of exogenous sex steroids, all patients were followed up until the point of full pubertal development (Tanner stage G4+ or B4+) to ensure that development did not arrest when off treatment.

Families of the patients with DP were invited to participate, with information about medical history and pubertal timing obtained by structured interviews and from archived height records. The criteria for DP in probands’ family members were one or more of: (1) age at takeoff; (2) PHV occurring 1.5 SD beyond the mean, that is, age at takeoff exceeding 12.9 and 11.3 years, or age at PHV exceeding 14.8 and 12.8 years in males and females; or (3) age at attaining adult height more than 18 or 16 years in males and females, respectively (19). Previous linkage analysis from this cohort did not find evidence for linked families sharing chromosomal segments identical by descent, suggesting that a founder effect is unlikely to be responsible for this phenotype (19).

Written informed consent was obtained from all participants. The study protocol was approved by the Ethics Committee for Pediatrics, Adolescent Medicine and Psychiatry, Hospital District of Helsinki and Uusimaa (extended to encompass Kuopio, Tampere, and Turku University Hospitals) (570/E7/2003). United Kingdom ethical approval was granted by the London-Chelsea NRES Committee (13/LO/0257). The study was conducted in accordance with the guidelines of the Declaration of Helsinki.

### Genetic analysis

**Figure 1.**
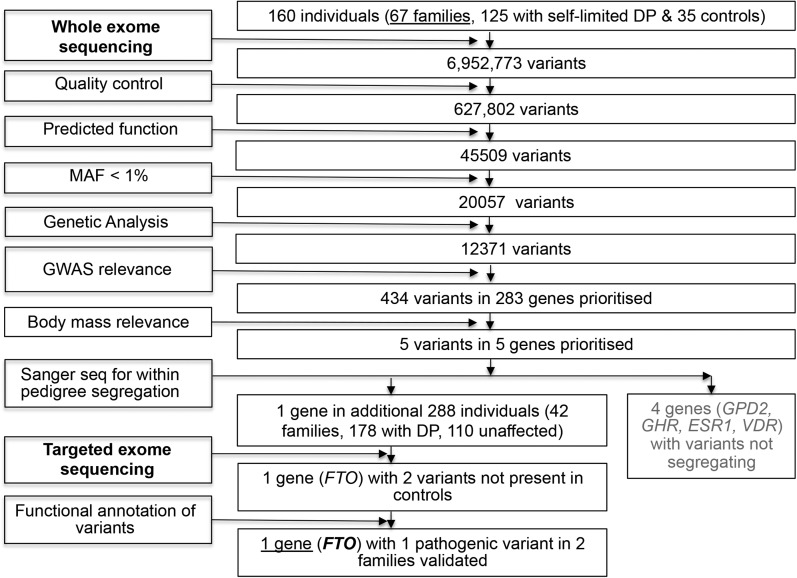
Flowchart of WES filtering strategy to identify candidate genes. WES was initially performed on DNA extracted from peripheral blood leukocytes of 160 individuals from the 67 most extensive families from our cohort (125 with DP and 35 controls), with exome capture on a Nimblegen v2 or Agilent v5 platform and sequencing on the Illumina HiSeq 2000. The exome sequences were aligned to the University of California, Santa Cruz hg19 reference genome. Picard tools and the genome analysis toolkit were used to mark PCR duplicates, realign around indels, and recalibrate quality scores and call variants. Variants were then analyzed further and filtered for potential causal variants using filters for quality control, predicted functional annotation, minor allele frequency (MAF), segregation with trait, and GWAS relevance (see *Materials and Methods* for further information on filtering criteria). Targeted exome sequencing using a Fluidigm array of a candidate gene identified after filtering was then performed in a further 42 families from the same cohort (288 individuals, 178 with DP and 110 controls). Variants after targeted resequencing were filtered using the same criteria as the WES data. Functional annotation of the variants is as described elsewhere in *Materials and Methods*.

Genetic analysis was performed in 160 individuals from the 67 most extensive families from our cohort with DP. These included 67 probands (male, n = 57; female, n = 10), 58 affected family members (male, n = 36; female, n = 22), and 35 unaffected family members (male, n = 13; female, n = 22). Whole-exome sequencing (WES) was performed on DNA extracted from peripheral blood leukocytes. Variants were analyzed and filtered for potential causal variants in Ingenuity Variant Analysis (Qiagen) using filters for quality control, predicted functional annotation, minor allele frequency (MAF), and GWAS relevance (Fig. 1). GWAS relevance filtering allowed identification of those remaining variants that lay within genes in linkage disequilibrium with 106 GWAS loci associated with AAM (n = 760) (5). Filters for genes implicated in body mass regulation were applied using a biological context filter with pathway analysis. Variants were filtered for segregation with trait in family members using conventional Sanger sequencing.

Targeted exome sequencing using a Fluidigm array of the remaining candidate gene identified postfiltering was then performed in a further 42 cohort families (288 individuals, 178 with DP [male, n = 106; female, n = 69) and 110 controls (male, n = 55; female, n = 58); Fig. 1]. Whole-gene rare variant burden testing was performed after sequencing.

### Growth pattern analysis

The pattern of prepubertal growth in the individuals carrying *FTO* variants was analyzed by using five screening parameters: (1) height for age SD score (HSDS); (2) BMI (calculated as weight in kilograms divided by height in meters squared) for age SDS (BMI SDS); (3) HSDS distance from target height (target height formula = 0.791 × mean parental HSDS − 0.147 for girls and 0.886 × mean parental HSDS − 0.071 for boys; (4) change in HSDS; and (5) change in BMI SDS across time with free age intervals. The calculations of the age-specific and sex-specific normal values for change in HSDS and change in BMI SDS were based on longitudinal reference measurements (22). Normality of linear growth was tested by using auxological screening rules based on data from >70,000 healthy Finnish children (23).

### *In silico* analysis

The FTO experimentally solved structure (PDB identifier: 4cxx) was used to study the structural effect of FTO variants. The following interactions involved in protein stability were considered: (1) salt bridges; (2) hydrogen bonds (H-bond); and (3) disulfide bridge (S*–*S bridge). N-glycosylation sites were determined based on the consensus sequence Asn-X-Thr/Ser (where X indicates any amino acid, except proline). The DSSP program was used to calculate surface accessibility and Disopred3 (24) was used to predict disordered protein regions.

### Functional annotation of FTO mutant proteins

Cloning of wild-type (WT) human FTO cDNA into pET302/NT-His has been described previously (25). The p.Leu44Val and p.Ala163Thr point mutations were introduced using PCR-mediated mutagenesis (QuickChange II, Agilent Technologies) using primers FTO_L44V (forward, 5′-GAATTCTATCAGCAGTGGCAGGTGAAATATCCTAAACTAATTCT-3′, reverse, 5′-AGAATTAGTTTAGGATATTTCACCTGCCACTGCTGATAGAATTC-3′) and FTO_A163T (forward, 5′-CACAGCATCCTCATTAGTCTTCTCTTTGGCAGCAA-3′, reverse, 5′-TTGC-TGCCAAAGAGAAGACTAATGAGGATGCTGTG-3′) and verified by sequencing. An RNase-cleavage assay (26) was used to measure the demethylation activity of FTO on 3-methyl-uridine (3-meU). Recombinant WT and mutant FTO expression plasmids were transformed into *Escherichia coli* BL21-Gold (DE3) (Stratagene) and cultured in Luria-Bertani broth and 50 μg/mL carbenicillin. Expression of the cloned gene was induced by the addition of isopropyl-*β*-d-1-thiogalactopyranoside at 1 mM final concentration at 15°C for 4 hours. The cells were harvested and pellets resuspended in lysis buffer [50 mM HEPES-KOH (pH 8.0), 2 mM 2-mercaptoethanol, 5% glycerol, and 300mM NaCl] before digestion with lysozyme (1 mg/mL). The cleared lysate was supplemented with imidazole (final concentration 10 mM) before mixing with 1 mL of prewashed nickel-nitrilotriacetic acid beads (Qiagen). After binding for 1 hour in the cold, the mixture was washed with lysis buffer supplemented with increasing concentrations of imidazole. FTO was eluted with 2 ml of lysis buffer containing 250 mM imidazole. The eluate was concentrated with a 30 kDa molecular mass cut-off concentrator (Sartorius Stedim) with buffer changing to 20 mM HEPES-KOH (pH 8), 5% glycerol, and 50 mM NaCl. Purified proteins were snap-frozen and stored at −80°C. Protein purity was estimated by Coomassie blue staining after resolving by sodium dodecyl sulfate–polyacrylamide gel electrophoresis (4%–12% gradient gels; Invitrogen).

For the dose response of FTO on 3-meU demethylation, recombinant FTO proteins were assayed as previously described (26). Each protein, at different protein concentrations from 0 to 1000 nM, was assayed in a reaction containing 100 nM substrate, 75 μM Fe(NH_4_)_2_(SO)_2_, 300 μM 2-oxoglutarate, 2 mM ascorbate, 50 μg/mL bovine serum albumin, and 62.5 μg/μL RNase A in 50 mM Tris-HCl buffer at pH 7.0. Samples were prepared in duplicate in a dark flat-bottomed 96-well plate and the 6-carboxyfluorescein emission was measured for 30 minutes at a wavelength of 520 nm with excitation at 485 nm. The measurement was performed at room temperature (25°C) using a microplate reader (Infinite M1000, Tecan). WT FTO protein and catalytically inactive mutant p.Arg316Gln (R316Q) served as positive and negative controls, respectively.

### Mouse experiments

Fto-deficient mice were a gift from Prof. Roger Cox (Medical Research Council Harwell, Oxford, U.K.) and were genotyped as previously described (27). This research is regulated under the Animals (Scientific Procedures) Act 1986 Amendment Regulations 2012 following ethical review by the University of Cambridge Animal Welfare and Ethical Review Body. Animals were kept under controlled temperature (22°C) and a 12-hour light/12-hour dark schedule (lights on 07:00 am to 7:00 pm). Standard chow (Special Diet Services) and water were available *ad libitum*.

For the vaginal opening (VO) study, female *Fto* heterozygous mice (*Fto*^+/−^) (n = 45) and their WT littermates (n = 24) were taken from either a male *Fto* WT × female *Fto*^+/−^ cross or a male *Fto*^+/−^ × female *Fto* WT cross. From postnatal day 21 (day of weaning) all female mice were weighed and visual examination of the vagina was carried out by placing the mouse on top of a cage lid and lifting the tail vertically away from the body. No excessive force was involved. The first day of VO was recorded when a complete opening was observed.

For all experiments, data are expressed as the mean ± standard error of the mean (SEM). To determine statistical significance, we used the unpaired *t* test (two-tailed) using SPSS software (version 24). A *P* value < 0.05 was considered statistically significant.

## Results

### Variants in GWAS genes implicated in body mass were identified following exome sequencing in families with self-limited DP

WES performed in the 67 largest and best phenotyped families from our cohort [160 individuals: a total of 125 individuals with DP (male, n = 93; female, n = 32) and 35 controls (male, n = 13; female, n = 22)] identified 6,952,773 variants after quality control (Fig. 1). Filtering to identify high-quality, rare, predicted deleterious variants not present in control subjects selected 12,371 variants in 7,470 genes. Of these 7470 genes, 238 were found to be in linkage disequilibrium with a GWAS locus for timing of puberty, and 5 of these 238 were genes implicated in body mass regulation or growth by pathway analysis. Of these five genes, four (*GPD2*, *GHR*, *ESR1*, and *VDR*) were found to have only variants that did not segregate with the DP trait in family members.

The remaining candidate gene, *FTO* (ENSG00000140718, gene identification number 79068), has been previously described in the literature as involved in pathways of energy homeostasis and growth (5), and it is known to act as an Fe(II) 2-oxoglutarate–dependent dioxygenase to repair alkylated DNA and RNA by demethylation (26). *FTO* contributes to the regulation of energy balance, and thus to the regulation of body size and fat accumulation.

Two variants in *FTO* [NM_001080432.2: c.130C>G p.Leu44Val and NM_001080432.2: c.487G>A (rs145884431) p.Ala163Thr] were identified in three families from our cohort and found in one or fewer control subjects (rare variant burden testing adjusted *P* = 0.058). Both variants are rare (MAF < 0.2%) heterozygous missense variants and are predicted to be benign or tolerated by more than two of five prediction software tools.

### Families with potentially pathogenic FTO variants display autosomal-dominant inheritance of DP phenotype and low body mass

**Figure 2.**
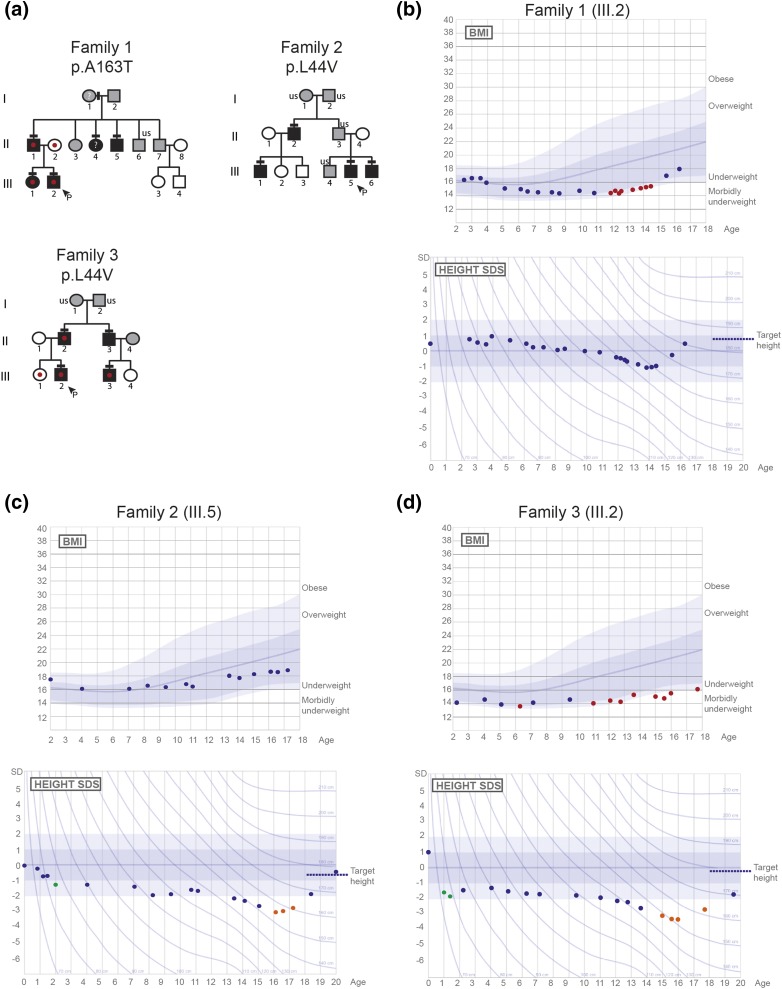
Pedigrees and auxological data of the families with potentially pathogenic *FTO* variants. (a) Squares indicate male family members; circles indicate female family members. Black symbols represent clinically affected, gray represent unknown phenotype, and clear symbols represent unaffected individuals. The arrowhead with “P” indicates the proband in each family, and “us” indicates unsequenced due to lack of DNA from that individual. The mutation in each family is given next to the family number; a horizontal black line above an individual’s symbol indicates that they are heterozygous for the variant as confirmed by either WES or Fluidigm array, and verified by Sanger sequencing. A red dot indicates that the individual was underweight (thinness grade 2 or more significant) and ‘?’ indicates that BMI information for that individual is not available. (b–d) BMI and HSDS charts for the probands of each of the three pedigrees (family 1.III.2, family 2.III.5, and family 3.III.2). Underweight values are shown in red, green dots indicate a significant deflection from previous height measurements, and orange dots indicate significant deflection from target height. Normal values, based on data from >70,000 healthy Finnish children, have been previously published (22).

The family identified with the p.Ala163Thr variant (family 1) and both of the families with the p.Leu44Val variant (families 2 and 3) displayed the typical autosomal inheritance pattern of the DP trait, with perfect segregation [Fig. 2(a)]. Affected individuals from family 1 with the p.Ala163Thr variant and from family 3 with the p.Leu44Val variant were particularly underweight in childhood, with the two probands from these families (individuals 1.III.2 and 3.III.2) falling into the thinness grade 2 category (28) before puberty [Fig. 2(b) and 2(d)]. Although there was some variability in this phenotype, all family members carrying *FTO* variants had age- and sex-adjusted BMI values in the lower range (<23) (Fig. 2; Supplemental Figs. 1–3; Table 1). Additionally, both of the probands from families 2 and 3 who carry the p.Leu44Val displayed faltering growth in early childhood. Both displayed significant deflection from previous height measurements in the 2 years following birth, as well as height significantly below target height in later adolescence associated with delayed pubertal growth [Fig. 2(c) and 2(d)] (22).

**Table 1.** **Clinical Data of Probands With FTO Variants**

**Case**	**Sex**	**Amino Acid Alteration**	**HSDS at** **Age 4 Years**	**HSDS at** **Age 8/9 Years**	**HSDS at** **Age 18 Years**	**Age- and Sex-Adjusted BMI at Age 18 Years**
1.II.1	M	p.Ala163Thr	—	1.1	1.7	16.9
1.III.2 (P)	M	p.Ala163Thr	1.1	0.5	1.1	17.1
1.III.1	F	p.Ala163Thr	0.9	1.0	1.1	17.3
1.II.5	M	p.Ala163Thr	−1.0	−1.0	−0.4	—
2.III.5 (P)	M	p.Leu44Val	−0.9	−1.4	−1.5	18.8
2.III.6	M	p.Leu44Val	−1.1	−1.3	—	—
2.II.2	M	p.Leu44Val	—	−0.8	−0.8	20.5
2.III.1	M	p.Leu44Val	0	−1.4	—	—
3.II.2	M	p.Leu44Val	—	−1.0	−0.9	18.6
3.III.2 (P)	M	p.Leu44Val	−0.9	−1.1	−1.3	18.7
3.II.3	M	p.Leu44Val		−0.4	−0.1	22.7
3.III.3	M	p.Leu44Val	−0.1	0.2	0.5	17.8

Height is expressed as SDS for national reference data for Finland at 4 years of age and at either 8 years for girls or 9 years for boys. Normal limits: change in HSDS <1.21, distance to target height at 4 years <1.76, distance to target height at 8/9 years <1.72 (22). P, proband.

### In silico analysis of potential mutations

We carried out *in silico* analysis using the solved structure of FTO (PDB identifier: 3lfm) to determine the possible pathogenicity of the identified variants. The hydrophobic residue leucine 44 is part of a solvent-exposed *α* helix on the surface. Substitution with valine is not predicted to alter the structure of FTO or interaction with iron molecules or DNA. However, L44 and other residues in the same solvent-exposed *α* helix form a motif (Supplemental Figs. 4 and 5), which is highly conserved across placental mammals but not reptiles, birds, or fish (Supplemental Fig. 6). This motif (residues 36 to 48) forms a patch on the FTO protein surface (Supplemental Fig. 7). This may act as a mammal-specific interaction site (between FTO and another protein) required for FTO function, for example, in reproductive development. In this scenario, a small change in side chain volume, such as leucine-to-valine, may have a subtle effect in protein–protein interaction and lead to a change in FTO activity *in vivo*.

Alanine 163 is a hydrophobic, not highly conserved residue (Supplemental Fig. 8A). Alanine 163 is at the end of the H4 *α* helix and the beginning of a long, disordered region (Supplemental Fig. 8B), which connects helices H4 and H5 (Supplemental Fig. 8C).

### FTO p.Leu44Val mutant protein displays reduced demethylase activity *in vitro*

**Figure 3.**
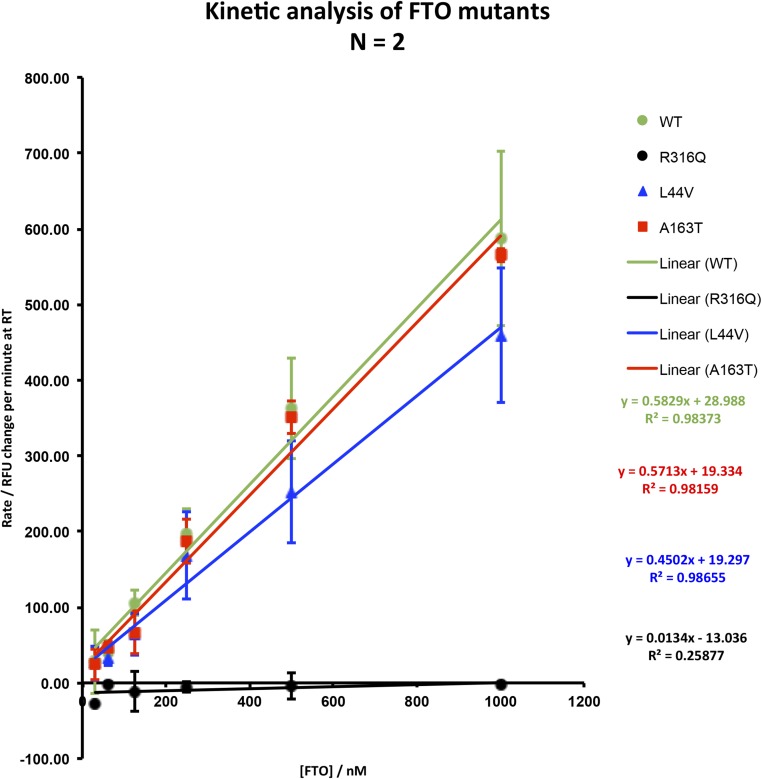
Demethylation assay assessing kinetic activity of mutant vs WT FTO proteins. FTO activity is proportional to the concentration present in the reaction. Demethylase activity is likely to be related to the ability of FTO to function as a sensor for cellular metabolism (29). The R316Q mutant is enzymatically dead across all concentrations tested. The A163T and L44V mutants showed demethylase activity toward methylated uridine in a dose-dependent manner but with different affinities.

We carried out functional characterization of the identified mutant FTO proteins (p.Leu44Val and p.Ala163Thr) as compared with WT protein. A previously verified RNase-cleavage assay was used to measure the demethylation activity of FTO on 3-meU (26). Although kinetic activity of the mutant protein p.Ala163Thr did not vary from WT using this assay, mutant protein p.Leu44Val showed an ∼20% lower kinetic activity than WT activity (Fig. 3) (29).

### FTO deficiency *in vivo* results in delayed VO in mice

**Figure 4.**
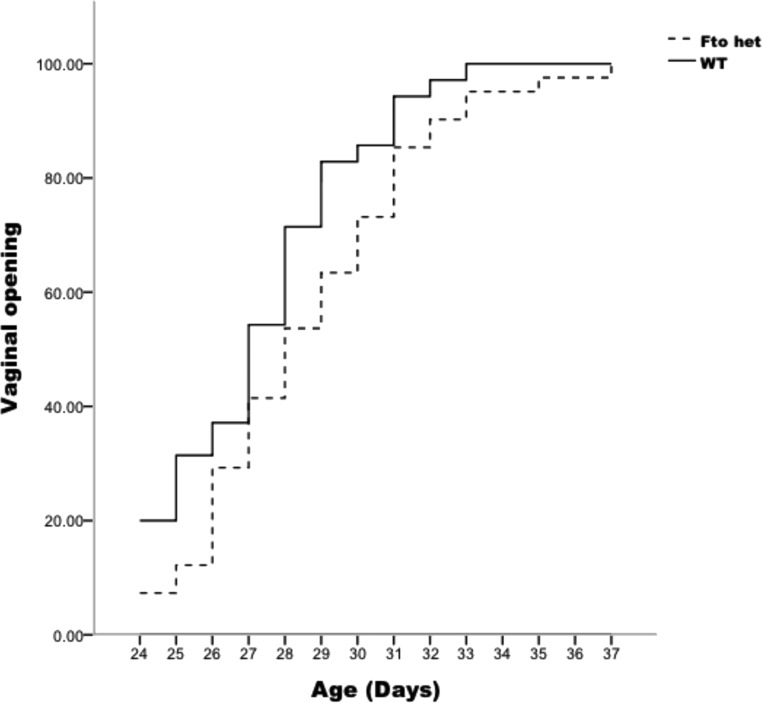
Timing of VO in WT and *FTO*^+/−^heterozygous mice. Cumulative percentages of mice displaying VO by postnatal day are shown for WT and *FTO*^+/−^ mice. WT mice, n = 24; *FTO*^+/−^ mice, n = 45. *P* < 0.05 by unpaired *t* test. het, heterozygous.

**Figure 5.**
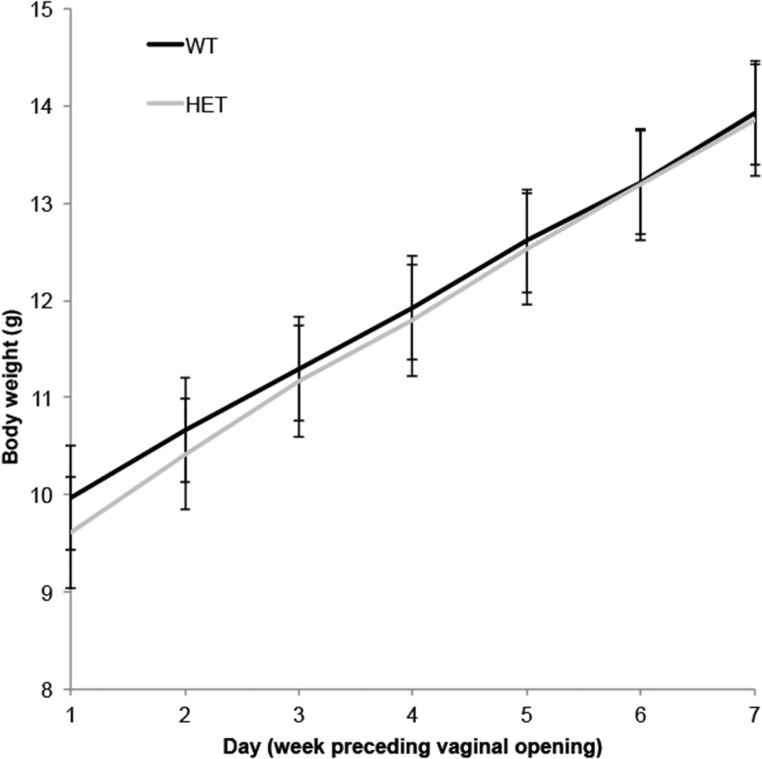
Mean body weight (grams) for WT and *Fto^+/−^* (heterozygous) mice in 7 days prior to VO. Mean body weight (grams) ± SEM: 11.64 ± 0.21 in WT mice (n = 24) vs 11.45 ± 0.14 in *Fto*^+/^ mice (n = 45). *P* = 0.467 by unpaired *t* test. Error bars show SEM for each group each day. HET, heterozygous.

To examine the influence of FTO activity on pubertal timing in an *in vivo* model, we examined timing of puberty in mice deficient for FTO in the heterozygous state (*Fto*^+/−^), in keeping with the human genotype identified. *Fto^−^*^/−^ mice were not selected for these experiments because of their poor postnatal health (30). *Fto*^+/−^ mice had significantly delayed timing of VO [mean postnatal day ± SEM: 27.20 ± 0.44 in WT (n = 24) vs 28.56 ± 0.48 in *Fto*^+/^ mice (n = 45), *P* = 0.047], an event that reflects the pubertal rise in estradiol (31) (Fig. 4). Mean body weight of the *Fto*^+/^ group was not significantly different from the WT mice [mean body weight (in grams) ± SEM: 11.64 ± 0.21 in WT vs 11.45 ± 0.14 in *Fto*^+/^ mice, *P* = 0.467] (Fig. 5).

Using simple linear modeling, the *Fto* genotype of the pup (heterozygous vs WT) explained ∼3% of the total variation in timing of VO. Consideration of an additional factor, maternal genotype, improved the model by increasing the significance of the association between pup genotype and timing of VO slightly (*P* = 0.04), and accounted for 6% of the total variation in timing of VO. In contrast, paternal genotype decreased the significance and total variation accounted for by the model.

## Discussion

GWAS of AAM in the general population have attempted to unravel the complex conundrum of which genetic factors influence the timing of puberty. Despite many loci being identified, clear evidence for the role of particular genes and pathways is for the most part lacking. Those genes lying within pathways of energy metabolism and growth appear promising, with the discovery of the role of *Lin28B* in *Caenorhabditis elegans* development (3) and the importance of leptin as a permissive signal in triggering the onset of puberty (9, 32).

The inheritance of DP is known to be under strong genetic influence with commonly an autosomal-dominant inheritance pattern, and thus represents a useful basis for the investigation of puberty genetics. Notably, self-limited or constitutional DP is often associated with slow maturation throughout childhood, implicating growth and energy metabolism pathways in its pathogenesis. Previously, genes in such pathways identified through GWAS have not been screened in patients with DP.

Our results have identified variants in *FTO* as a potential contributory factor in the development of self-limited DP in three pedigrees from our large cohort of patients with familial DP. *FTO* was the first obesity-susceptibility gene identified through GWAS and continues to be the locus with the largest effect on BMI and obesity risk (10). Those DP patients identified with *FTO* variants from our study showed reductions in body mass. The *FTO* variants carried by our DP patients may result in reduced fat mass, which would in turn contribute to a delay in the timing of pubertal onset. This delay may be mediated directly through reduced leptin levels. Although we do not routinely measure leptin levels in DP patients, leptin levels have been shown to be significantly lower in pubertal-age patients with self-limited DP (33).

Notably, in an *in vivo* model, *Fto^+/−^* mice had a significantly delayed onset of puberty as compared with WT mice. In the 7 days preceding puberty onset, however, body weight was not significantly different between the two pup genotype groups. Previous studies have demonstrated that *Fto*^−/−^ mice show a 30% to 40% reduction in body weight by 6 weeks of age (30) and that transgenic mice with additional copies of *Fto* show a dose-dependent increase in body and fat mass (34). However, the relationship between *FTO* genotype, fat mass, and leptin levels remains somewhat unclear. *Fto*-deficient mice do become obese when subjected to a high-fat diet, although they remain sensitive to the anorexigenic effects of leptin (30, 35).

Moreover, it is possible that *FTO* gene dosage may have an effect on energy homeostasis independent of effects on fat mass (34), including on the balance between catabolic and anabolic pathways (36). *FTO* has been identified as an amino acid sensor acting, via mTOR, to influence appropriate levels of development and translation (29). *FTO* is expressed within the hypothalamus in several sites critical for energy balance, including in the arcuate nucleus within proopiomelanocortin neurons (37, 38). In one study *Fto* levels in the arcuate nuclei of fasted mice fell by up to 60%, and this was not rescued by leptin administration. Other studies have shown conflicting results in the effects on *Fto* messenger RNA levels of fasting, depending on whether whole hypothalamus or arcuate nucleus was studied and on the length of fast (38). However, *Fto*^−/−^ mice display blunted starvation-induced Npy messenger RNA induction (30). More recent studies have suggested that Fto may influence the metabolic outcomes of a high-fat diet via hypothalamic signaling pathways acting independently of body weight (35). Mutations in *FTO*, including those with greatly reduced demethylase activity (*e.g.*, pR316Q, Fig. 3), have been identified in human subjects associated with both lean and obese phenotypes (25). We were not able in our study to identify the mechanism by which the p.Ala163Thr variant might affect protein function; although no reduction in demethylation activity was demonstrated, it is possible that this variant may produce a deleterious effect by another route, for example by defects in posttranslational modification or protein degradation.

Thus, FTO may be important for signaling energy sufficiency and the “healthy energy balance” required for pubertal onset. Our *in silico* analysis suggests that the p.Leu44Val mutation we have identified may represent a mammal-specific interaction site between FTO and another protein (or DNA), important for FTO function in terms of reproductive development. Moreover, maternal genotype may contribute to pubertal timing, as demonstrated from our *Fto^+/−^* mice data. A reproductive phenotype present in *Fto* heterozygote mothers could expose pups to a suboptimal environment that could influence their puberty timing.

Finally, our finding of maturational delay in growth in early childhood in the two probands with p.Leu44Val mutation is of interest. Constitutional delay in growth is seen in a subset of patients with DP, and our findings implicate mutations in energy pathway genes in the pathogenesis of patients with such a phenotype.

Overall, our discovery of two rare variants in *FTO* associated with self-limited DP in our large familial cohort, and of delayed VO in *FTO*-deficient mice, provides evidence that perturbations in pathways of energy homeostasis and growth may potentially produce a phenotype of DP. We note that despite this extensive analysis, only 3 of 67 probands were identified with potentially pathogenic variants in such pathways, highlighting the high degree of heterogeneity in the genetic basis of self-limited DP. These findings merit further exploration in our own cohort and in other populations, including subgroup analysis of DP patients with low BMI from early childhood.
