# Supplementary material for: Contributions of Function-Altering Variants in Genes Implicated in Pubertal Timing and Body Mass for Self-Limited Delayed Puberty
Source: J Clin Endocrinol Metab. 2017 Nov 16;103(2):649–59. doi: 10.1210/jc.2017-02147 (PMC5800831; doi:10.1210/jc.2017-02147)
Supplement: Supplementary file 1 [file jc.2017-02147.sd1.pdf]

Supplementary Data

Contributions of function-altering variants in genes implicated in pubertal timing and body mass for self-limited delayed puberty

Sasha R Howard, Leonardo Guasti, Ariel Poliandri, Alessia David, Claudia P Cabrera, Michael R Barnes, Karoliina Wehkalampi, Stephen O'Rahilly, Catherine E Aiken, Anthony P Coll, Marcella Ma, Debra Rimmington, Giles SH Yeo, Leo Dunkel

Table of Contents

Supplementary Methods: Patient details and Genetic Analysis

Supplementary Fig. 1: Auxological data of the family members from Family 1 with potentially pathogenic *FTO* variant.

Supplementary Fig. 2: Auxological data of the family members from Family 2 with potentially pathogenic *FTO* variant.

Supplementary Fig. 3: Auxological data of the family members from Family 3 with potentially pathogenic *FTO* variant.

Supplementary Fig. 4: Surface representation of FTO bound to 3---methylthymidine.

Supplementary Fig. 5: 3D structure of FTO bound to 3---methylthymidine and iron (PDB 3flm).

Supplementary Fig. 6: Multiple sequence alignment between human FTO and its orthologues.

Supplementary Fig. 7: Tertiary structure of FTO local to L44 residue

Supplementary Fig. 8: p.A163T sequence and structural analysis.

Supplementary Table 1: Genes involved in energy metabolism and growth pathways implicated in the timing of puberty in the general population from genome wide association studies

Supplementary Methods:

Patient Details

For family members diagnosis of DP was based on PHV occurring 1.5 SD beyond the mean, i.e. age at takeoff exceeding 12.9 and 11.3 yr, or age at PHV exceeding 14.8 and 12.8 yr in males and females. This 1.5SD cutoff has sensitivity of 98% in identifying boys with Tanner stages G2 later than 14.0 years and sensitivity of 97% in identifying girls with Tanner stage B2 later than 13.0 years.

Genetic Analysis

Genetic analysis was performed in 160 individuals from the 67 most extensive families from our cohort with DP. These included 67 probands (male n=57, female n=10), 58 affected family members (male n=36, female n=22) and 35 unaffected family members (male, n=13, female n=22). Whole exome sequencing (WES) was performed on DNA extracted from peripheral blood leukocytes, using a Nimblegen V2 or Agilent V5 platform and Illumina HiSeq 2000 sequencing. The exome sequences were aligned to the UCSC hg19 reference genome. Picard tools and the genome analysis toolkit were used to mark PCR duplicates, realign around indels, recalibrate quality scores and call variants.

Variants were analyzed and filtered for potential causal variants in Ingenuity Variant Analysis (Qiagen) using filters for quality control, predicted functional annotation, minor allele frequency (MAF), and GWAS relevance (Figure 1). Quality control included thresholds for call quality, read depth and upstream pipeline filtering. Predicted functional annotation involved prioritizing nonsense, exonic missense, splice site variants, structural or promoter

changes, or variants deleterious to a microRNA. Filtering by MAF entailed including those variants with minor allele frequency (MAF) <1% in the 1000 Genomes database, the NHLBI exome variant server, and ExAC and gnoMAD databases. GWAS relevance filtering allowed identification of those remaining variants that lay within genes in linkage disequilibrium with 106 GWAS loci associated with AAM[1]. All genes in linkage disequilibrium with these GWAS AAM loci (using inclusive limits: D' > 0.8; r^2: no limit) were selected using the Broad institute SNAP tool (SNP annotation and proxy search). Linkage disequilibrium data was calculated using Haploview 4.0, based on phased genotype data from the International HapMap Project and the 1000 Genomes Project. A total of 760 genes were selected using this SNAP tool, and 'GWAS relevance filtering' allowed identification of those remaining variants that lay within these 760 genes[2]. Filters for genes implicated in body mass regulation were applied using a biological context filter with pathway analysis. Variants were then filtered for segregation with trait: variants present in ≥ n-1 affected individuals (where n = number of affected individuals in a given pedigree) and not present in more than one unaffected individual being retained. Family members were screened using conventional Sanger sequencing.

Targeted exome sequencing using a Fluidigm array of the remaining candidate gene identified post-filtering was then performed in a further 42 families from our cohort (288 individuals, 178 with DP; male=106, female=69 and 110 controls; male=55, female=58, Figure 1). Variants post targeted re-sequencing were filtered using the same criteria as the WES data: quality

control, predicted functional annotation, minor allele frequency and segregation with trait.

Whole gene rare variant burden testing was performed post sequencing. Fisher's exact test was used to compare the prevalence of deleterious variants in our cohort with the Finnish population, using the ExAC Browser (Exome Aggregation Consortium (ExAC), Cambridge, MA: http://exac.broadinstitute.org, accessed September 2015). All variants from the ExAC database with minor allele frequency <1%, predicted to be deleterious by Polyphen-2 [3] or SIFT [4], were included in the analysis. A multiple comparison adjustment was applied post hoc using the Benjamini & Hochberg method [5], as detailed in [6]. Variants were confirmed via Sanger sequencing.

Supplementary Figures

Family 1 (p.A163T)

Family 1 (II.5)

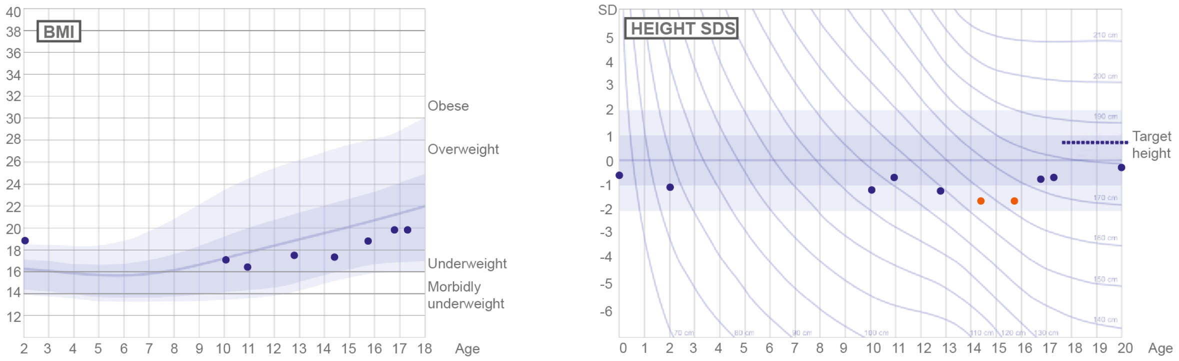

Family 1 (III.1)

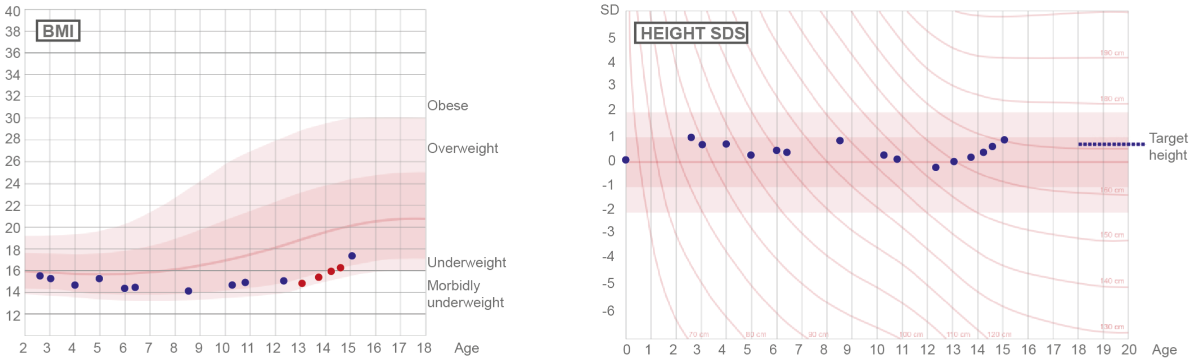

Supplementary Fig. 1 - Auxological data of the family members from Family 1 with potentially pathogenic *FTO* variant.
BMI and height standard deviation score (SDS) charts for the family members with *FTO* variant. Underweight values are shown in red, green dots indicate a significant deflection from previous height measurements and orange dots indicate significant deflection from target height. Normal values, based on data from >70,000 healthy Finnish children, have been previously published [7].

Family 2 (p.L44V)

Family 2 (III.6)

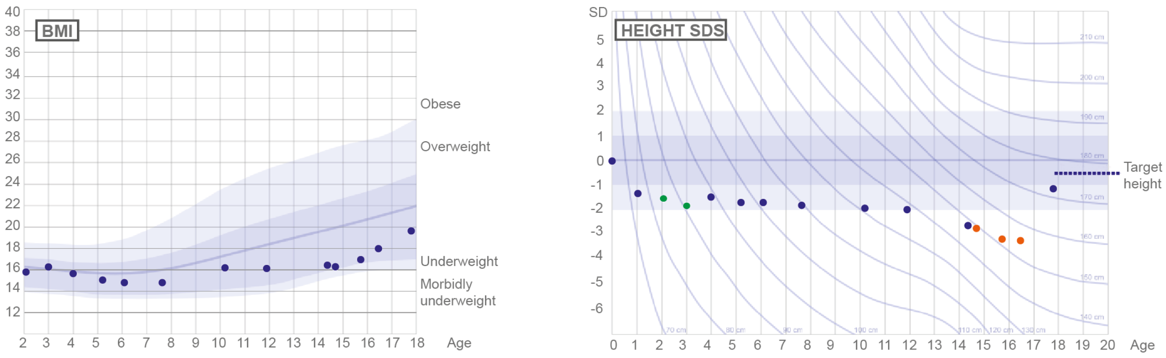

Family 2 (II.2)

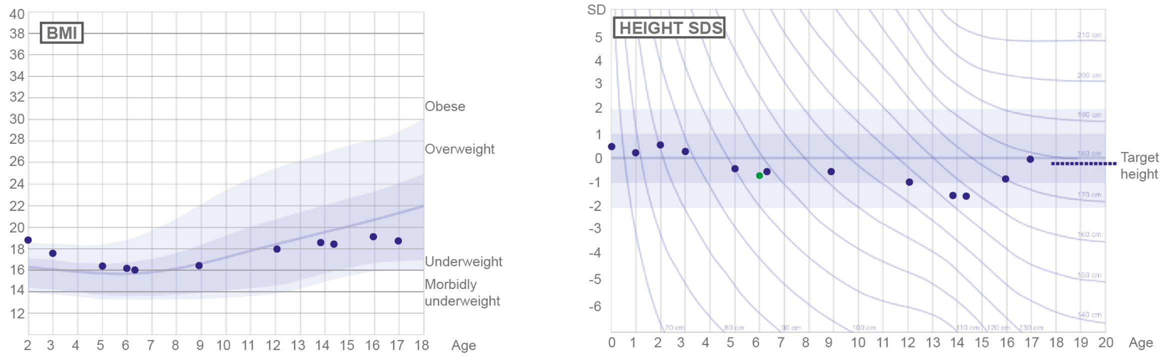

Supplementary Fig. 2 - Auxological data of the family members from Family 2 with potentially pathogenic *FTO* variant.
BMI and height standard deviation score (SDS) charts for the family members with *FTO* variant. Underweight values are shown in red, green dots indicate a significant deflection from previous height measurements and orange dots indicate significant deflection from target height. Normal values, based on data from >70,000 healthy Finnish children, have been previously published [7].

Family 3 (p.L44V)

Family 3 (II.2)

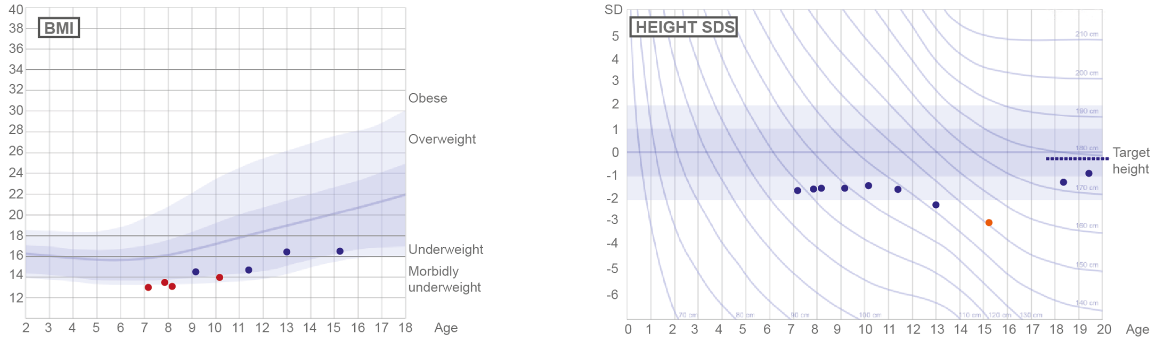

Family 3 (II.3)

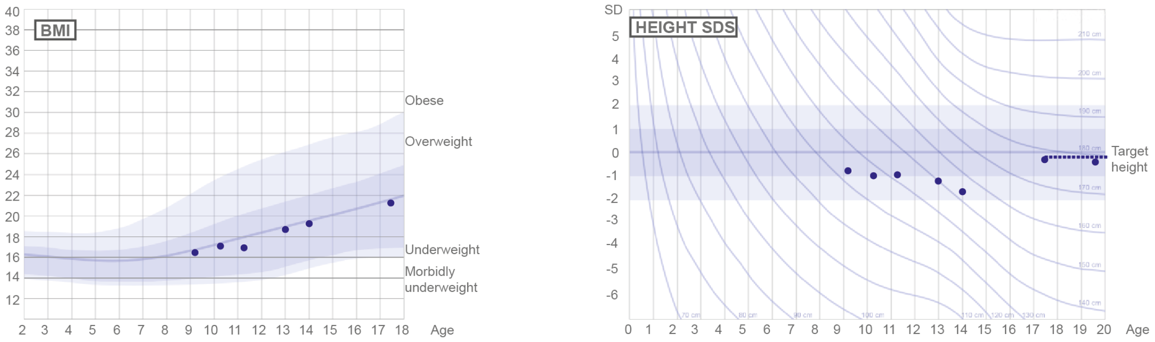

Family 3 (III.3)

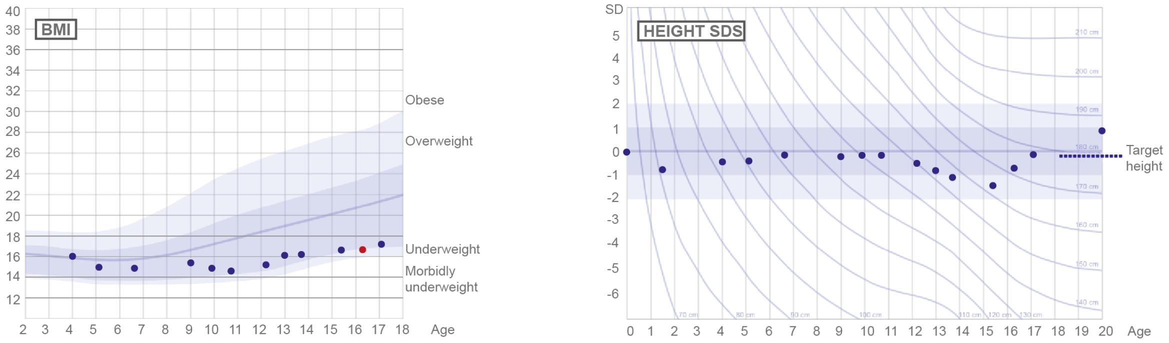

Supplementary Fig. 3 - Auxological data of the family members from Family 3 with potentially pathogenic *FTO* variant.
BMI and height standard deviation score (SDS) charts for the family members with *FTO* variant. Underweight values are shown in red, green dots indicate a significant deflection from previous height measurements and orange dots indicate significant deflection from target height. Normal values, based on data from >70,000 healthy Finnish children, have been previously published [7].

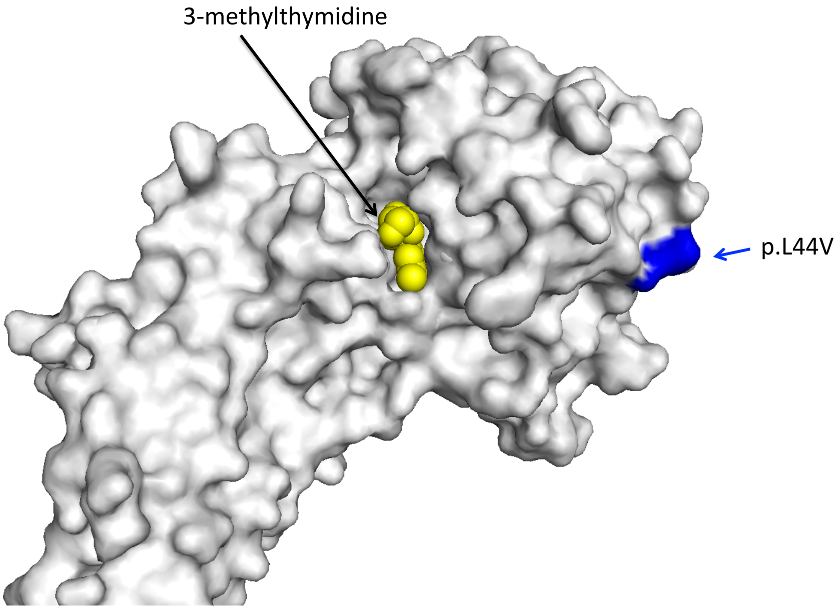

Supplementary Fig. 4 - Surface representation of FTO (here presented in grey) bound to 3---methylthymidine (in yellow). Leucine 44 is presented in blue.

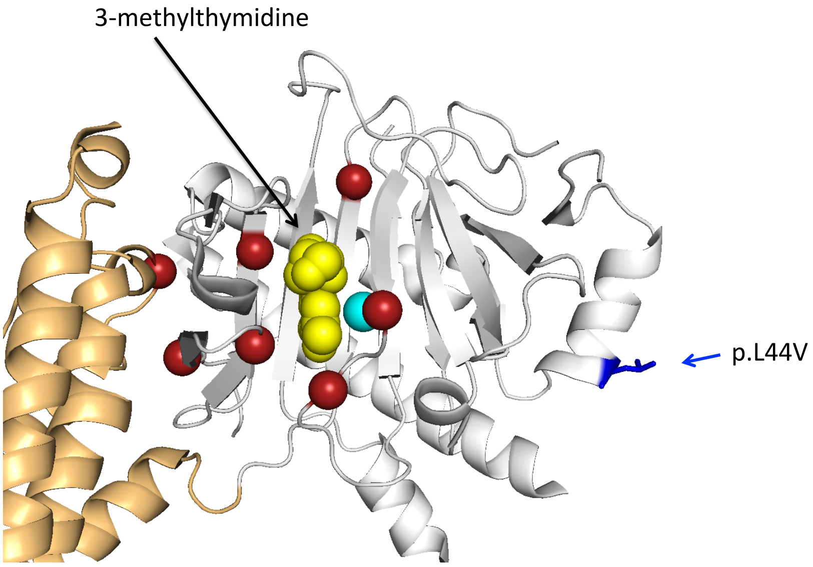

Supplementary Fig. 5 - 3D structure of FTO bound to 3---methylthymidine and iron (PDB 3flm)
FTO N---terminal domain is presented in grey and the C---terminal domain in orange. Dna is presented as yellow spheres, iron as a blue sphere. Deleterious mutations p.R96M or W, p. Y108A, p.F114D, p.E234P, p.C392D are presented as red spheres. p.L44V is presented in blue.

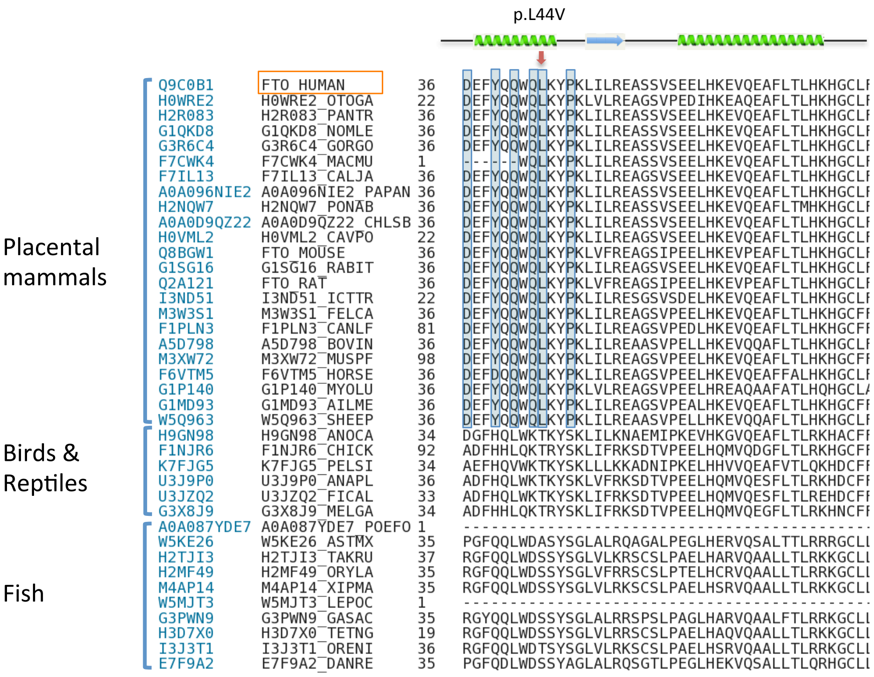

Supplementary Fig. 6 – Multiple sequence alignment between human FTO and its orthologues.
Orthologous sequences from other species were retrieved from Ensembl. Species are classified in “Placental mammals”, “Birds & Reptiles” and “Fish” according to Ensembl classification. For each species, FTO Uniprot accession number (in blue) and entry name (in black) are presented at the beginning of the row. The position of deleterious mutations on the human FTO sequence is indicated by a red arrow. L44 and other surrounding residues part of the same alpha helix form a motif, which is highly conserved across placental mammals but not in reptiles, birds and fish.

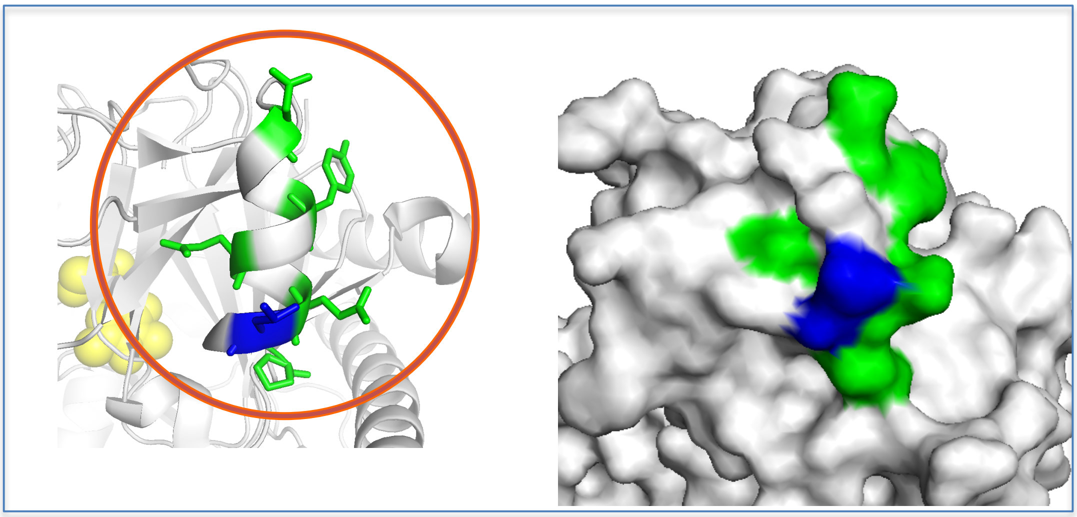

Supplementary Fig. 7 – Tertiary structure of FTO local to L44 residue. Residue L44 is presented in blue and other residues located on the same alpha helix and conserved across placental mammals are presented in green. FTO structure is shown as cartoon on the left and as surface on the right. 3---methylthymidine is presented as yellow spheres in the cartoon representation.

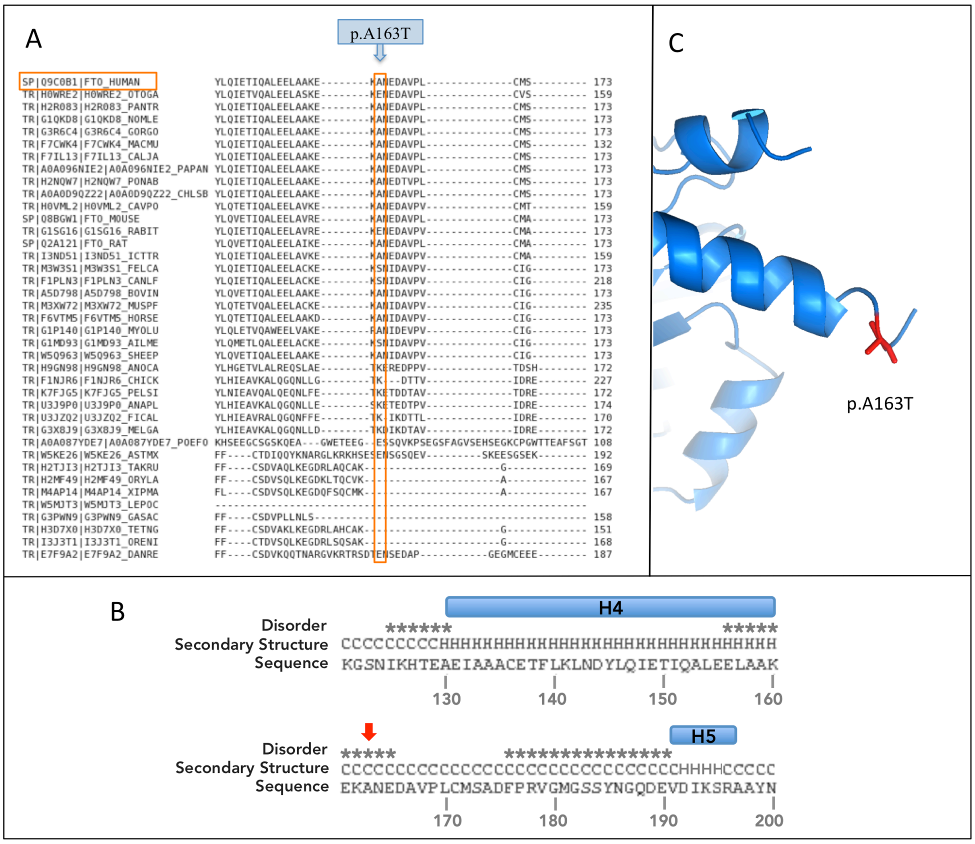

Supplementary Fig. 8 - p.A163T sequence and structural analysis.
Panel A: a multiple sequence alignment shows that alanine at position 163 is not conserved. Panel B: the position of Ala163 (indicated by a red arrow) is presented in relation to FTO secondary structure (H, alpha helix; C, residues that are not part of an alpha helix or a beta strand). Residues predicted to be disordered are indicated with an asterisk. Panel C: FTO tertiary structure. Ala163 (presented in red) is at the end of FTO alpha helix H4 and is not predicted to disrupt FTO function. FTO structure is visualized using visualisation program (http://www.pymol.org/).

	Gene Symbol	
FTO	*GNPDA2*	*LEKR1*
SEC16B	*BDNF*	*DLK1*
TMEM18	*LIN28B*	*LEPR-LEPROT*
NEGR1	*SIX6*	
TNN13K	*CENPW-NCO7*	

Supplementary Table 1 – Genes involved in energy metabolism and growth pathways implicated in the timing of puberty in the general population from genome wide association studies (adapted from Perry et al [1]).

References

1. Perry JR, Day F, Elks CE, Sulem P, Thompson DJ, Ferreira T, He C, Chasman DI, Esko T, Thorleifsson G, Albrecht E, Ang WQ, Corre T, Cousminer DL, Feenstra B, Franceschini N, Ganna A, Johnson AD, Kjellqvist S, Lunetta KL, McMahon G, Nolte IM, Paternoster L, Porcu E, Smith AV, Stolk L, Teumer A, Tsernikova N, Tikkanen E, Ulivi S, Wagner EK, Amin N, Bierut LJ, Byrne EM, Hottenga JJ, Koller DL, Mangino M, Pers TH, Yerges-Armstrong LM, Hua Zhao J, Andrulis IL, Anton-Culver H, Atsma F, Bandinelli S, Beckmann MW, Benitez J, Blomqvist C, Bojesen SE, Bolla MK, Bonanni B, Brauch H, Brenner H, Buring JE, Chang-Claude J, Chanock S, Chen J, Chenevix-Trench G, Collee JM, Couch FJ, Couper D, Coviello AD, Cox A, Czene K, D'Adamo A P, Davey Smith G, De Vivo I, Demerath EW, Dennis J, Devilee P, Dieffenbach AK, Dunning AM, Eiriksdottir G, Eriksson JG, Fasching PA, Ferrucci L, Flesch-Janys D, Flyger H, Foroud T, Franke L, Garcia ME, Garcia-Closas M, Geller F, de Geus EE, Giles GG, Gudbjartsson DF, Gudnason V, Guenel P, Guo S, Hall P, Hamann U, Haring R, Hartman CA, Heath AC, Hofman A, Hooning MJ, Hopper JL, Hu FB, Hunter DJ, Karasik D, Kiel DP, Knight JA, Kosma VM, Kutalik Z, Lai S, Lambrechts D, Lindblom A, Magi R, Magnusson PK, Mannermaa A, Martin NG, Masson G, McArdle PF, McArdle WL, Melbye M, Michailidou K, Mihailov E, Milani L, Milne RL, Nevanlinna H, Neven P, Nohr EA, Oldehinkel AJ, Oostra BA, Palotie A, Peacock M, Pedersen NL, Peterlongo P, Peto J, Pharoah PD, Postma DS, Pouta A, Pylkas K, Radice P, Ring S, Rivadeneira F, Robino A, Rose LM, Rudolph A, Salomaa V, Sanna S, Schlessinger D, Schmidt MK, Southey MC, Sovio U, Stampfer MJ, Stockl D, Storniolo AM, Timpson NJ, Tyrer J, Visser JA, Vollenweider P, Volzke H, Waeber G, Waldenberger M, Wallaschofski H, Wang Q, Willemsen G, Winqvist R, Wolffenbuttel BH, Wright MJ, Australian Ovarian Cancer S, Network G, kConFab, LifeLines Cohort S, InterAct C, Early Growth Genetics C, Boomsma DI, Econs MJ, Khaw KT, Loos RJ, McCarthy MI, Montgomery GW, Rice JP, Streeten EA, Thorsteinsdottir U, van Duijn CM, Alizadeh BZ, Bergmann S, Boerwinkle E, Boyd HA, Crisponi L, Gasparini P, Gieger C, Harris TB, Ingelsson E, Jarvelin MR, Kraft P, Lawlor D, Metspalu A, Pennell CE, Ridker PM, Snieder H, Sorensen TI, Spector TD, Strachan DP, Uitterlinden AG, Wareham NJ, Widen E, Zygmunt M, Murray A, Easton DF, Stefansson K, Murabito JM & Ong KK. Parent-of-origin-specific allelic associations among 106 genomic loci for age at menarche. *Nature* 2014 **514** 92-97.
2. Elks CE, Perry JR, Sulem P, Chasman DI, Franceschini N, He C, Lunetta KL, Visser JA, Byrne EM, Cousminer DL, Gudbjartsson DF, Esko T, Feenstra B, Hottenga JJ, Koller DL, Kutalik Z, Lin P, Mangino M, Marongiu M, McArdle PF, Smith AV, Stolk L, van Wingerden SH, Zhao JH, Albrecht E, Corre T, Ingelsson E, Hayward C, Magnusson PK, Smith EN, Ulivi S, Warrington NM, Zgaga L, Alavere H, Amin N, Aspelund T, Bandinelli S, Barroso I, Berenson GS, Bergmann S, Blackburn H, Boerwinkle E, Buring JE, Busonero F, Campbell H, Chanock SJ, Chen W, Cornelis MC, Couper D, Coviello AD, d'Adamo P, de Faire U, de Geus EJ, Deloukas P, Doring A, Smith GD, Easton DF, Eiriksdottir G, Emilsson V, Eriksson J, Ferrucci L, Folsom AR, Foroud T, Garcia M, Gasparini P, Geller F, Gieger C, Gudnason V, Hall P, Hankinson

SE, Ferreli L, Heath AC, Hernandez DG, Hofman A, Hu FB, Illig T, Jarvelin MR, Johnson AD, Karasik D, Khaw KT, Kiel DP, Kilpelainen TO, Kolcic I, Kraft P, Launer LJ, Laven JS, Li S, Liu J, Levy D, Martin NG, McArdle WL, Melbye M, Mooser V, Murray JC, Murray SS, Nalls MA, Navarro P, Nelis M, Ness AR, Northstone K, Oostra BA, Peacock M, Palmer LJ, Palotie A, Pare G, Parker AN, Pedersen NL, Peltonen L, Pennell CE, Pharoah P, Polasek O, Plump AS, Pouta A, Porcu E, Rafnar T, Rice JP, Ring SM, Rivadeneira F, Rudan I, Sala C, Salomaa V, Sanna S, Schlessinger D, Schork NJ, Scuteri A, Segre AV, Shuldiner AR, Soranzo N, Sovio U, Srinivasan SR, Strachan DP, Tammesoo ML, Tikkanen E, Toniolo D, Tsui K, Tryggvadottir L, Tyrer J, Uda M, van Dam RM, van Meurs JB, Vollenweider P, Waeber G, Wareham NJ, Waterworth DM, Weedon MN, Wichmann HE, Willemsen G, Wilson JF, Wright AF, Young L, Zhai G, Zhuang WV, Bierut LJ, Boomsma DI, Boyd HA, Crisponi L, Demerath EW, van Duijn CM, Econs MJ, Harris TB, Hunter DJ, Loos RJ, Metspalu A, Montgomery GW, Ridker PM, Spector TD, Streeten EA, Stefansson K, Thorsteinsdottir U, Uitterlinden AG, Widen E, Murabito JM, Ong KK & Murray A. Thirty new loci for age at menarche identified by a meta-analysis of genome-wide association studies. *Nat Genet* 2010 **42** 1077-1085.

3. Adzhubei IA, Schmidt S, Peshkin L, Ramensky VE, Gerasimova A, Bork P, Kondrashov AS & Sunyaev SR. A method and server for predicting damaging missense mutations. *Nat Methods* 2010 **7** 248-249.
4. Kumar P, Henikoff S & Ng PC. Predicting the effects of coding non-synonymous variants on protein function using the SIFT algorithm. *Nat Protoc* 2009 **4** 1073-1081.
5. Benjamini Y, Drai D, Elmer G, Kafkafi N & Golani I. Controlling the false discovery rate in behavior genetics research. *Behav Brain Res* 2001 **125** 279-284.
6. Howard SR, Guasti L, Ruiz-Babot G, Mancini A, David A, Storr HL, Metherell LA, Sternberg MJ, Cabrera CP, Warren HR, Barnes MR, Quinton R, de Roux N, Young J, Guiochon-Mantel A, Wehkalampi K, Andre V, Gothilf Y, Cariboni A & Dunkel L. IGSF10 mutations dysregulate gonadotropin-releasing hormone neuronal migration resulting in delayed puberty. *EMBO Mol Med* 2016.
7. Saari A, Harju S, Makitie O, Saha MT, Dunkel L & Sankilampi U. Systematic growth monitoring for the early detection of celiac disease in children. *JAMA Pediatr* 2015 **169** e1525.
